# Magnetic Targeting of Growth Factors Using Iron Oxide Nanoparticles

**DOI:** 10.3390/nano8090707

**Published:** 2018-09-10

**Authors:** Michal Marcus, Alexandra Smith, Ahmad Maswadeh, Ziv Shemesh, Idan Zak, Menachem Motiei, Hadas Schori, Shlomo Margel, Amos Sharoni, Orit Shefi

**Affiliations:** 1 Faculty of Engineering, Bar Ilan University, Ramat Gan 5290002, Israel; michalb87@gmail.com (M.M.); drmaswadeh@hotmail.com (A.M.); zivshem6@gmail.com (Z.S.); idan.zack@gmail.com (I.Z.); motiei.biu@gmail.com (M.M.); hadas.schori@gmail.com (H.S.); 2 Bar Ilan Institute of Nanotechnologies and Advanced Materials, Ramat Gan 5290002, Israel; alesmith07@gmail.com (A.S.); shlomo.margel@mail.biu.ac.il (S.M.); amos.sharoni@biu.ac.il (A.S.); 3 Department of Chemistry, Bar Ilan University, Ramat Gan 5290002, Israel; 4 Department of Neurosurgery, Sheba Medical Center, Ramat Gan 5290002, Israel; 5 Department of Physics, Bar Ilan University, Ramat Gan 5290002, Israel

**Keywords:** nerve growth factor, magnetic nanoparticles, neuronal regeneration, magnetic targeting, sciatic nerve injury

## Abstract

Growth factors play an important role in nerve regeneration and repair. An attractive drug delivery strategy, termed “magnetic targeting”, aims to enhance therapeutic efficiency by directing magnetic drug carriers specifically to selected cell populations that are suitable for the nervous tissues. Here, we covalently conjugated nerve growth factor to iron oxide nanoparticles (NGF-MNPs) and used controlled magnetic fields to deliver the NGF–MNP complexes to target sites. In order to actuate the magnetic fields a modular magnetic device was designed and fabricated. PC12 cells that were plated homogenously in culture were differentiated selectively only in targeted sites out of the entire dish, restricted to areas above the magnetic “hot spots”. To examine the ability to guide the NGF-MNPs towards specific targets in vivo, we examined two model systems. First, we injected and directed magnetic carriers within the sciatic nerve. Second, we injected the MNPs intravenously and showed a significant accumulation of MNPs in mouse retina while using an external magnet that was placed next to one of the eyes. We propose a novel approach to deliver drugs selectively to injured sites, thus, to promote an effective repair with minimal systemic side effects, overcoming current challenges in regenerative therapeutics.

## 1. Introduction

Effective nerve regeneration and repair following injury or neurodegenerative diseases is under an extensive study. Much research has been devoted to the development of novel therapeutics, drugs, and engineered platforms [1,2,3,4,5,6,7,8]. One of the main challenges is the ability to deliver these therapeutic agents selectively to the target injured sites, thus, to enhance drug accumulation and promote an effective repair with minimal systemic side effects [9,10,11]. A recent strategy, termed “magnetic targeting”, utilizes controlled magnetic fields to direct magnetic elements to selected target regions. By conjugating bioactive molecules to magnetic carriers, the complexes become sensitive to magnetic fields and therefore can be remotely guided [12,13,14,15]. Magnetic fields that are applied externally can be designed to attract the magnetic complexes to specific sites. This novel delivery approach has been recently demonstrated in several settings in different fields [16,17,18,19,20,21], e.g., the delivery of chemo- and phototherapeutics to cancerous tissues [22,23], the delivery of stem cells to injured sites [24,25].

Growth factors are fundamental components in nerve tissue development and repair, proposed as promising regenerative agents [26,27,28]. One leading factor is the nerve growth factor (NGF) [29], which is involved in neuronal differentiation, survival, and maintenance [28,30]. NGF demonstrates protective effects for nerve tissue inducing axonal regeneration. NGF bestows high pharmacological potential for the treatment of central and peripheral nerve injuries [31,32]. Additionally, NGF has been shown to play a therapeutic role in neurodegenerative diseases and specifically Alzheimer’s [33,34,35,36,37]. Many groups, including ours, have developed various non-magnetic strategies for local delivery of NGF. Nanostructured porous silicon chips were synthesized for prolonged controlled release of NGF for localized treatment [38]. Likewise, fibered nerve conduits with spatial gradients of neurotrophic factors were shown to release NGF locally at the injured site [39]. Lipid carriers encapsulating NGF were developed as well for delivery of the factor [40]. For magnetic targeting of NGF, several magnetic carriers have been developed and demonstrated in vitro. Micera et al. has developed magnetic microspheres loaded with NGF and demonstrated controlled release of the payload at targeted site [41]. Gilbert et al. combined NGF-releasing poly-l-lactic acid (PLLA) nanoparticles with magnetic nanoparticles to create NGF gradients within a tissue culture dish and successfully directed neurite extension [42].

An attractive type of magnetic carriers is of iron oxide nanoparticles. As iron oxide nanoparticles possess superparamagnetic properties, they are sensitive to magnetic manipulations only under the influence of magnetic fields. Iron oxide nanoparticles in the fully oxidized state (Fe_2_O_3_) are stable and they can be surface functionalized to prevent aggregation, improve cellular uptake and avoid cytotoxicity [43,44]. Moreover, it has been shown that bare iron oxide nanoparticles, without additional active compounds, show positive effects on neuronal cells [45]. Several formulations of iron oxide nanoparticles have already been approved by the Food and Drug Administration (FDA) as magnetic resonance imaging (MRI) contrast agents for clinical use arising the potential for additional medical applications [46]. Previously, we have shown that the conjugation of NGF to iron oxide nanoparticles extends NGFs’ half-life and consequently enhances NGF effect on cells. Inducing differentiation with NGF conjugated iron oxide nanoparticles significantly promoted neurite outgrowth and complexity of the neuronal branching trees, and enhanced molecular differentiation markers, in comparison to free NGF treatment [47]. Similar studies that linked other bioactive molecules, such as basic fibroblast growth factor (bFGF), glial cell-derived neurotrophic factor (GDNF), thrombin, to these nanoparticles, showed enhanced activity and stability as well [48,49,50].

Here, we covalently conjugated NGF to magnetic iron oxide nanoparticles (NGF-MNPs) and fabricated a modular magnetic device that can be used to apply the pre-designed profiles of magnetic fields. We used the controlled magnetic fields to deliver the NGF–MNPs complex to specific regions in vitro, demonstrating a selective activation of NGF within a culture plate. To demonstrate the targeting approach in vivo, we magnetically guided NGF-MNPs and demonstrated the accumulation of the particles at targeted tissues including mice sciatic nerve and retina. The ameliorate activity resulting from the linkage of factor to the nanoparticles, together with the ability to direct the nanoparticles to selected sites of interest, propose an improved treatment for patients that may lead to better regeneration outcomes of the nervous system. This method may be useful for delivering other agents presenting a promising magneto-therapeutic route.

## 2. Materials and Methods

### 2.1. Synthesis and Characterization of NGF-Conjugated Nanoparticles

Maghemite magnetic nanoparticles were synthesized and conjugated to NGF according to previous publications [47,51] with slight modifications. In brief, MNPs were prepared by nucleation, followed by controlled growth of six thin films of *γ*-Fe_2_O_3_ onto the gelatin-RITC/iron oxide nuclei (RITC i.e., Rhodamine B Isothiocyanate, Sigma Aldrich, Rehovot, Israel). MNPs were coated with human serum albumin (HSA) by the precipitation of the protein onto the surface of the MNPs adding 0.2 mg of HSA per milligram of MNPs dispersion. Then, MNPs were encapsulated by polyethylene glycol (PEG) terminated with *N*-Hydroxysuccinimide (NHS) through the linking of amine and/or hydroxyl groups of the HSA. The covalent conjugation of NGF to MNPs was accomplished via the interaction of the amine and/or hydroxyl groups of the growth factor with the terminal activated NHS groups of PEG on nanoparticle's surface. 125 µL of a NGF solution (0.4 mg/mL, pH 7.4) was added to 125 µL of the PEG-activated MNPs that were dispersed in bicarbonate buffer (3 mg/mL, pH 8.4) at a MNPs/NGF weight ratio of 30. The reaction mixture of the PEG-activated MNPs was then shaken at 4 °C for 20 min. Blocking of the residual NHS was then accomplished by adding 1% glycine (*w*/*v*) and then shaking for an additional hour. The amount of NGF conjugated to the MNPs is 3.3 µg/mg MNPs. The concentration of the NGF conjugated to the PEG-MNPs was determined by measuring the unbound NGF with a mouse IgG ELISA kit (ChemiKine Nerve Growth Factor Sandwich ELISA Kit, Chemicon International, Temecula, CA, USA) and subtracting it from the initial concentration. The reported values are an average of three measurements. Transmission electron microscopy (TEM) pictures were obtained with a Tecnai C2 Biotwin electron microscope with 120 kV accelerating voltage (FEI, Hillsboro, OR, USA). Samples for TEM were prepared by placing a drop of the diluted sample on a 400-mesh carbon-coated copper grid. The hydrodynamic diameter was measured while using a particle analyzer, Zetasizer Nano ZS (Malvern Panalytical, Malvern, UK). The reported hydrodynamic diameter corresponds to the Z-average value. Fluorescence excitation and emission spectra measurements were performed on a Perkin-Elmer LS-50B digital fluorimeter (Norwalk, CT, USA). Magnetic measurements of nanoparticles were performed at room temperature, using SQUID Magnetometer (Quantum Design, San Diego, CA, USA). Particle solution was placed in a suitable squid capsule, and was left to dry for 48 h. Then, the capsule was capped and placed in the squid holder. A known mass of particles was measured and the results were normalized to 1 g.

### 2.2. Magnetic Stand Preparation and Magnetic Field Simulation

The device was designed by “Autodesk 123D Design” software (2016 Version, Autodesk Inc., San Rafael, CA, USA). The beehive device was designed as a 72 mm × 55 mm × 20 mm (length × width × height) block with pinholes of 1.2 mm diameter. The distance between 2 adjacent pinhole centers was defined as 4 mm. The device was fabricated while using a three-dimensional (3D) printer (FORMLAB2, Formlabs Inc., Somerville, MA, USA) and made of clear tough polymer resin with a resolution of 0.05 µm. The magnetic flux density resulting from the different geometries was calculated by means of numerical field calculations while using the software Comsol Multiphysics 4.1 (Comsol Multiphysics GmbH, Goettingen, Germany).

### 2.3. Cell Culture

PC12 cells were grown in suspension in the Roswell Park Memorial Institute (RPMI) 1640 medium supplemented with 10% horse serum (HS), 5% fetal bovine serum (FBS), 1% l-glutamine, 1% penicillin–streptomycin, and 0.2% amphotericin, in a humidified incubator at 37 °C containing 5% CO_2_ (medium and supplements were purchased from Biological Industries, Beit Haemek, Israel). To induce differentiation, cells were seeded on plates coated with collagen type 1 and incubated overnight in serum reduced medium (1% HS). Culture dish wash placed on magnet stand, and NGF-MNPs were added to the medium. For fluorescent visualization of localized cellular uptake, after 24 h of incubation, the cells were washed three times to remove excess particles and were observed by confocal microscopy. For the localized differentiation experiments, the cells were incubated with NGF-MNPs for four days.

### 2.4. Cell Viability Assay

The tetrazolium dye based assay, XTT (Biological Industries, Beit Haemek, Israel), was used for a quantitative assessment of the cell viability. 10,000 cells were seeded on 96-well plates and incubated with NGF-MNPs at concentrations ranging from 10–250 µg/mL. After 24 h XTT reaction solution was added to the wells and incubated for 5 h at 37 °C. Absorbance was measured at 450 nm (630 nm background) while using a spectrophotometer (BioTek Synergy 4, Winooski, VT, USA). The absorbance signal of the particles at the same tested concentrations was measured and subtracted from measurements.

### 2.5. In Vivo Experiments

All of the experimental procedures and methods were approved by the Animal Care Committee of Bar-Ilan University (approval number: 30042018) and were performed in accordance with the National Institutes of Health guidelines and regulations. Balb/c mice (*n* = 3 in each test group (MNPs IV + Magnet/MNPs IV/control)) were purchased from Envigo (Rehovot, Israel).

The mid-thigh incision method was used for harvesting the sciatic nerve. A small (~5.0 mm) vertical incision was made along the thigh using scissors and the skin was retracted laterally. The muscles of the posterior thigh were split to expose the sciatic nerve. The nerve was gently lifted using a non-magnetic forceps. 10 µL of magnetic particles (4 mg/mL, dispersed in sodium bicarbonate buffer (0.84% in DDW, Ph = 8.3)), were injected while using U 100 Insulin Syringe. Magnetic tip (1 Tesla) was placed above the sciatic nerve ~0.5 cm from injection site. After five minutes, the sciatic nerve was extracted and transferred into a solution of PFA + Sucrose (4%). The magnet that was used in the experiment was composed of a magnetic tip placed on top of a cylindrical magnet. The magnetic tip was made from Hiperco 50A to ASTM A801 type 1, shaped as truncated cone with 18 mm base and 0.5 mm tip with total height of 18 mm. The cylindrical magnet (Metal Suppliers Online LLC, Hampstead, NH, USA) is axially magnetized, made of NdFeB N50, coated with nickel cooper nickel, with 18 mm in diameter and length of 18 mm.

In i.v. injection experiments, balb/c mice were i.v. injected with NGF-MNPs (4 mg/mL, 100 µL for each mouse). A cylindrical magnet (0.5 Tesla) was placed near right eye for 30 min. At the end of the experimental period the mice were given a lethal dose of pentobarbitone (170 mg/kg). Their eyes were enucleated, and the retinas were detached and extracted into a solution of PBS. For the bio-distribution profile, six organs (kidney, liver, spleen, brain, lungs, and eye) were extracted from all test groups immediately after magnetic treatment. For histology, the organs were extracted 18 days after MNPs injection. Tissue samples were embedded in paraffin block and were cut using a microtome to 3 µm sections. For H&E staining the samples were deparaffinized, rehydrated, and immersed in H&E stain. Then, the sections were dehydrated and mounted on slides.

### 2.6. Prussian Blue Staining

For Prussian blue staining (Iron Stain Kit, ScyTek Laboratoreis, Logan, UT, USA), Potassium Ferrocyanide solution (5%) was mixed with an equal volume of Hydrochloric Acid solution (2%). Samples were incubated in mixed solution for 15 min and washed with distilled water.

### 2.7. ICP Analysis

Tissues samples were dissolved in nitric acid solution on a heat block. Samples were diluted with deionized water to a final volume of 5 mL and filtered. The iron concentration was determined while using inductively coupled plasma optical emission spectroscopy (ICP-OES, SPECTRO ARCOS ICP-OES, FHX22 MultiView plasma). A calibration curve with known iron concentrations was prepared and the iron concentration was determined according to absorbance values, as compared to calibration curves. All of the samples were analyzed by ICP under the same experimental conditions.

### 2.8. Imaging

In vitro fluorescent imaging was performed while using a Leica TCS SP5 confocal microscope with an Acousto-Optical Beam Splitter (Leica Microsystems, Wetzlar, Germany). In vivo fluorescent imaging was performed by the Maestro II in vivo imaging system, 2D planar fluorescence imaging of small animals (Cambridge Research and Instrumentation, Inc., Woburn, MA, USA). A Green excitation/emission filter set was used for our experiments (λ_ex_, 525–547 nm; λ_em_ > 560 nm). The Liquid Crystal Tunable Filter (LCTF, Cambridge Research and Instrumentation, Inc., Woburn, MA, USA) was programmed to acquire image cubes from λ = 560–620 nm with an increment of 10 nm per image. H&E stained slides were imaged using Axio Imager microscope (Zeiss, Oberkochen, Germany).

## 3. Results and Discussion

### 3.1. NGF-MNPs: Synthesis, Characterization and Interactions with Cells

**Figure 1:**
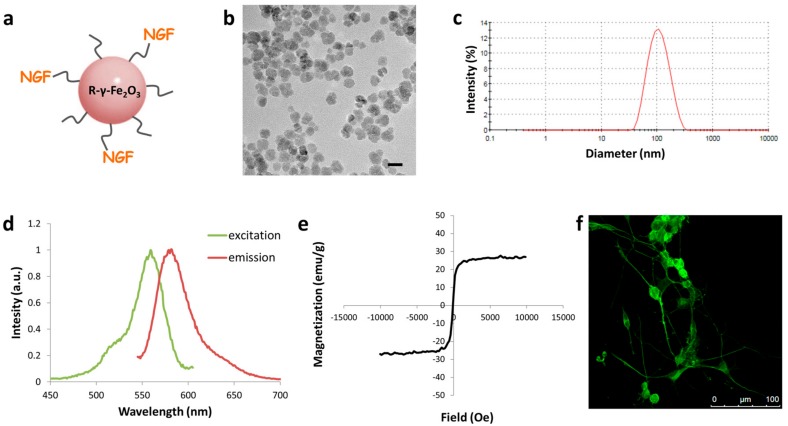
(**a**) Schematic illustration of nerve growth factor to iron oxide nanoparticles (NGF-MNPs); (**b**) Transmission electron microscopy image of NGF-MNPs. Scale bar = 50 nm; (**c**) Dynamic light scattering measurement of NGF-MNPs hydrodynamic diameter; (**d**) Fluorescence spectra of NGF-MNPs; (**e**) Magnetization curve of NGF-MNPs at room temperature; (**f**) PC12 cells four days after induction of differentiation by NGF-MNPs.

Magnetic iron oxide nanoparticles that were labelled with the fluorescent probe rhodamine were synthesized and covalently conjugated to NGF through a spacer arm of polyethylene glycol (Figure 1a). Size and uniformity of the particles were characterized while using transmission electron microscopy imaging. TEM images demonstrate spherical particles with an average diameter of 18 ± 4 nm (Figure 1b). The hydrodynamic diameter of the NGF–MNPs, as determined by dynamic light scattering is 99 ± 47 nm (Figure 1c), whereas neat MNPs present a diameter of 45 ± 17 (Supplementary Materials, Figure S1). The difference in diameter size verifies NGF conjugation to particles. Fluorescence spectra of NGF-MNPs, measured by fluorimeter, reveal an excitation peak at 559 nm and an emission peak at 579 nm in water (Figure 1d). These results show that rhodamine fluorescent properties remain, and that the particle synthesis process does not interfere with the fluorophore’s spectrum. Magnetic measurements show that the *M*(*H*) curve of the NGF-MNPs has no hysteresis loop, indicating the superparamagnetic behavior of these particles (Figure 1e). The saturation magnetization of the NGF-MNPs is ~27 emu/g. The saturation magnetization of bulk maghemite is about 76 emu/g [52]. The difference between the values derives from the fact that the NGF-MNPS are not pure maghemite, and they are composed of additional materials (i.e., gelatin, Rhodamine Isothiocyanate).

In order to determine whether the conjugated NGF protein retained its biological activity, and was not harmed by the binding process, we examined NGF-MNPs’ functionality. We used PC12 cells, a common model for neuronal differentiation, which differentiate and extend branching neurites when exposed to NGF in vitro [53]. NGF-MNPs were added to PC12 cells that were cultured a day before on collagen-coated plates. The NGF-MNPs were found to induce PC12 differentiation. The cells demonstrated neurite outgrowth and they formed a branched network (Figure 1f).

**Figure 2:**
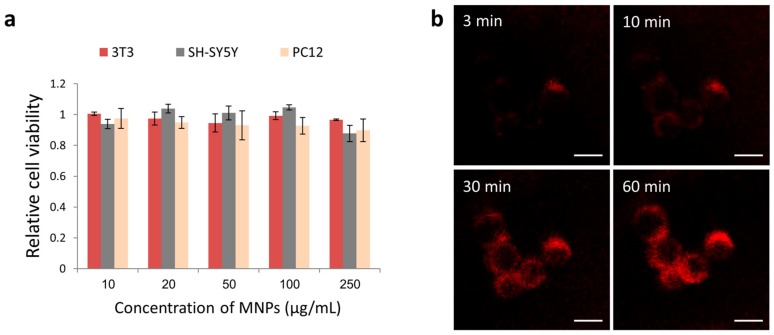
(**a**) XTT viability assay of 3T3, SH-SY5Y, and PC12 cells incubated with various concentrations of NGF-MNPs after 24 h (*n* = 3); (**b**) Fluorescent images PC12 cells incubated with NGF-MNPs at different time points. Scale bar = 10 µm.

Although several formulations of iron oxide nanoparticles have already been approved by the FDA as MRI contrast agents for clinical use [54,55], other iron oxide nanoparticles show toxic effects [56,57]. Therefore, the toxicity level of the particles must be tested. Cell viability XTT assay showed that the NGF-MNPs exhibited no significant cytotoxicity to several different types of cells, including rat adrenal pheochromocytoma cells (PC12), human neuroblastoma cells (SH-SY5Y), and mouse embryonic fibroblast cells (NIH 3T3), even under high concentrations up to 250 µg/mL (Figure 2a). Cellular uptake of NGF-MNPs was studied by time-lapse fluorescence microscopy. Already three minutes after the NGF-MNPs addition to cell culture dish, a red fluorescence signal was detected from cytoplasm of PC12 cells (Figure 2b). The fluorescence increased in a time-dependent manner.

### 3.2. In Vitro Magnetic Targeting of NGF-MNPs

**Figure 3:**
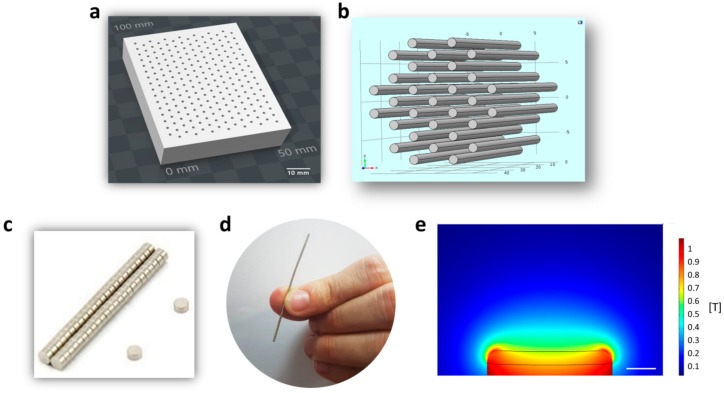
(**a**) A beehive block design illustration; (**b**) Magnetic rods ordered in hexagons grid; (**c**) Circular neodymium magnets piled to rods; (**d**) Size illustration of magnetic rods; (**e**) Simulation of magnetic flux density in COMSOL software. The image presents a side view of magnetic flux density of magnet rod. Intensity is color coded (low intensity in dark blue, high intensity in red). Scale bar = 0.25 mm.

For in vitro local targeting of the NGF-MNPs we designed a 3D platform that can hold portable magnets in a modular combination according to need. This modular magnetic device enables the creation of magnetic “hot spots” that attract the magnetic carriers. Furthermore, such modular magnetic devices can be used for many magnet-based applications, including applying mechanical stress on membranes, cytoskeleton, or other organelles regulating gene expression and intracellular processes. For example, Zablotskii et al. used a hexagonal matrix consisting of millimetre sized cylinders to magnetically regulate cellular nanomechanics of stem cells [58]. Here, we printed a beehive shaped non-magnetic block embedded with parallel holes (Figure 3a). This non-magnetic device was designed as a plastic porous rectangular prism. The pinholes in the device are scattered equally, as the distance between two adjacent pinhole centers is equal (Figure 3b). By arranging the magnetic rods at a desired form within the block, specific patterns of external magnetic fields can be achieved above. We used neodymium magnets N42 (NdFeB) with cylinder dimensions of 1 mm diameter and 1 mm height each (Figure 3c). Piling these magnets one on top of the other creates the rod structure (Figure 3d). We calculated the effective magnetic field above one single magnetic rod that can serve as a building block for any chosen configuration. Figure 3e presents the simulated magnetic flux density that was generated by a single magnet rod. 

**Figure 4:**
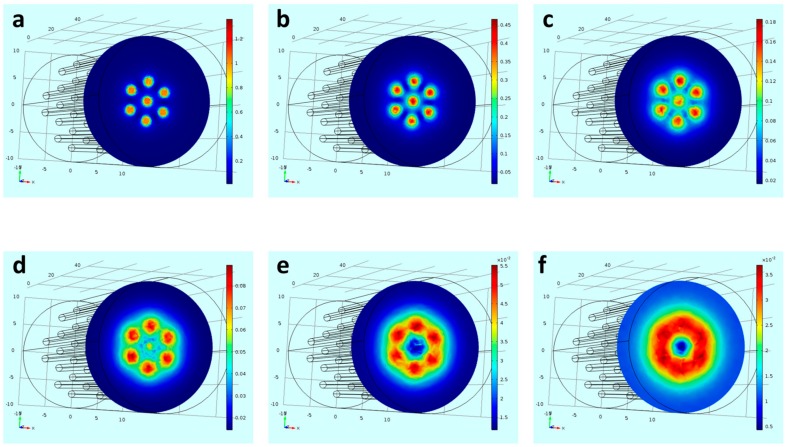
Simulated magnetic flux density (**a**) 0.1 mm from top (**b**) 0.5 mm from top (**c**) 1 mm from top (**d**) 1.5 mm from top (**e**) 2 mm from top (**f**) 2.5 mm from top. Color scale bar in [T].

Figure 4 presents an example of a circular arrangement of magnets. Six pinholes in the hive device were filled with the neodymium magnet rods in a form of a circle with a single rod in the center. The six magnets of the perimeter were polarized in one direction and the center magnet was polarized in the opposite magnetization direction. We simulated the magnetic flux density at different planes: 0.1 mm, 0.5 mm, 1 mm, 1.5 mm, 2 mm, and 2.5 mm above the magnetic rods. Simulation of the magnetic flux density one mm above the stand (equals to the thickness of the plastic plate culture) shows a maximal intensity value of ~0.16 T above the magnetic rods, appearing as seven magnetic “hot spots”. It can be seen that, according to the simulation, 2.5 mm above the device surface the highest magnetic intensity is shaped as a continuous circle forming an effective magnetic attractive area of a ring. Magnetic field gradients, which are responsible for MNPs actuation at a distance, were simulated demonstrating the effective magnetic area above the device (Figure S2).

**Figure 5:**
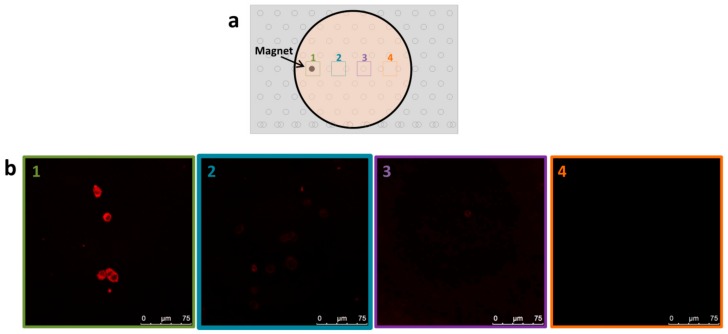

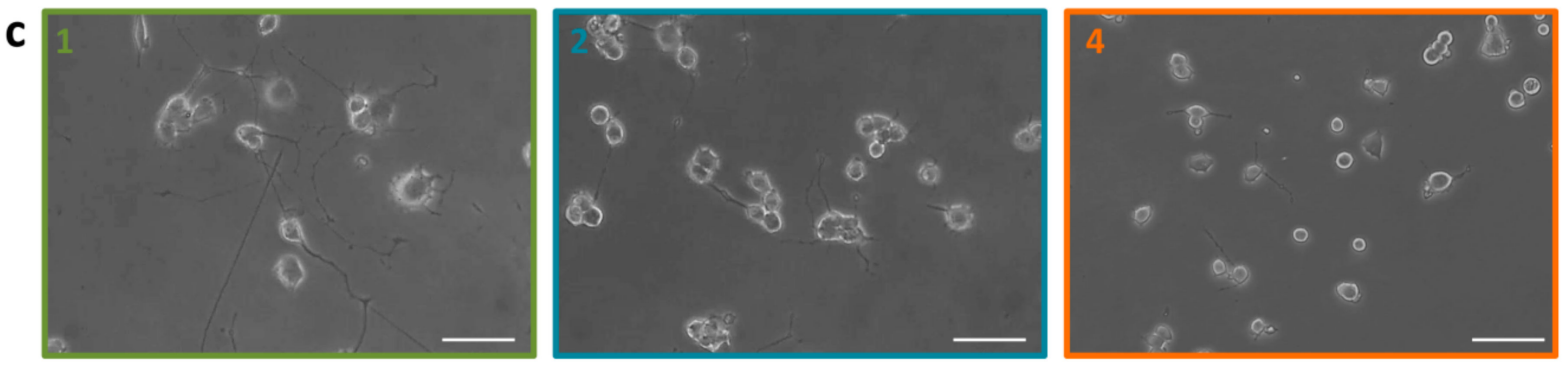
(**a**) Schematic illustration of cell culture dish with magnet location. Areas 1, 2, 3, and 4 indicate sites of imaging; (**b**) Confocal fluorescent images of PC12 cells at different locations relative to magnet, as marked in A; (**c**) Phase contrast images of differentiated PC12 cells at different locations relative to magnet, as marked in A. Scale bar = 50 µm.

In order to induce differentiation locally, we used the 3D platform of the beehive block embedded with the magnetic rods to design “hot spots” that will attract the NGF-MNPs selectively to specific regions in culture dish. PC12 cells were cultured overnight and they were shown to be spread homogenously in the culture. To demonstrate clearly the localized effect, the culture dish was placed atop the magnetic stand and only a single magnet rod was placed in one of the pinholes (Figure 5a). NGF-MNPs were added to the culture dish for incubation, and before imaging cells were washed, therefore only nanoparticles interacting with cells could be detected. It can be seen that cells above magnet site demonstrate a high uptake of the NGF-MNPs, whereas cells in other areas of the dish, away from the magnet, show no accumulation of nanoparticles (Figure 5b).

To examine the ability to induce efficient differentiation locally, the NGF-MNPs that were added to the homogenous cell culture were incubated for four days before imaging. Phase contrast images of the PC12 cells, far and close to the magnetic “hot spots”, indicate the localized differentiation effect (Figure 5c). PC12 cells above magnetic site were at an advanced stage of differentiation with long neurites interconnecting between neighboring cells. The longest neurite observed was >250 µm. On the contrary, cells that were 0.5 cm away from magnet site had shorter neurites, with the longest neurite of ~70 µm. The cells located far from the magnet site (zone 4) showed no differentiation.

The nonhomogeneous levels of differentiation result from the magnetic attracting forces that localize the factors activity only at selected areas. Due to the superparamagnetic behavior of the magnetic particles, NGF-MNPs are magnetized only in the presence of an external magnetic field and are attracted towards the magnetic sites by the magnetic field gradients (according to theoretical equations elaborated in Supplementary 3). Magnets with other strength and sizes will lead to other differentiation profiles. As was previously reported, the conjugated NGF to the magnetic carriers is functional and induces even higher levels of differentiation. We have shown that the conjugated factor is uptaken by the cells through the normal pathway [47].

### 3.3. In Vivo Magnetic Targeting of NGF-MNPs

**Figure 6:**
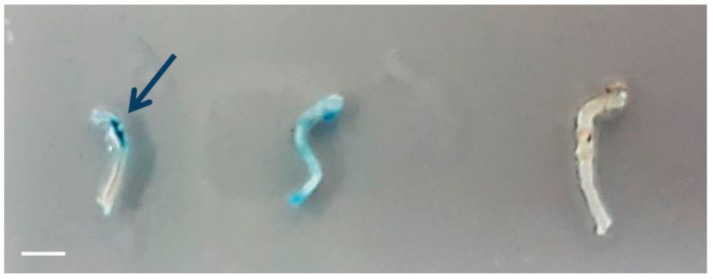
In vivo magnetic targeting in sciatic nerve model. Images of extracted sciatic nerves: **Left**: nerve injected with MNPs. A magnet tip was placed externally following injection, leading to MNPs accumulation near magnet (stained in blue). **Middle**: nerve injected with MNPs, without an external magnet. MNPs are distributed along the nerve. **Right**: Control (no injection of MNPs). Scale bar = 4 mm.

To demonstrate the potential of the magnetic targeting approach for NGF therapy, we used two common in vivo models to direct the magnetic nano-carriers towards specific target sites in the nervous system. First, we injected the NGF-MNPs directly to the sciatic nerve and showed the ability to localize particles along the nerve by an external magnet (see methods). We used a magnetic tip that generates a strong localized magnetic flux and placed it above the sciatic nerve 0.5 cm away from the injection site. We applied the magnetic field for five minutes and then extracted the nerves. To study the distribution of MNPs along the sciatic nerve, Prussian blue staining was used. Figure 6 shows a clear accumulation of MNPs close to the magnet tip demonstrating their flow along the sciatic nerve following the injection. Thus, magnetic targeting can be useful for directing drugs and active factors towards damaged sites along nerves. The method can also help to maintain the drugs in desired locations for a prolonged effect as was also proposed in other studies [59]. The magnetic delivery approach presents a promising way to induce regeneration not only in cut injuries but also in cases of nerve crush that do not require a nerve conduit or autografts, which is currently an unsolved pathology [60].

**Figure 7:**
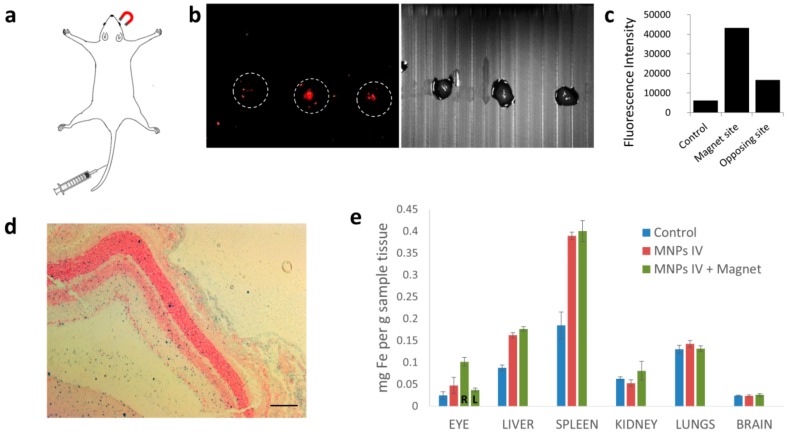

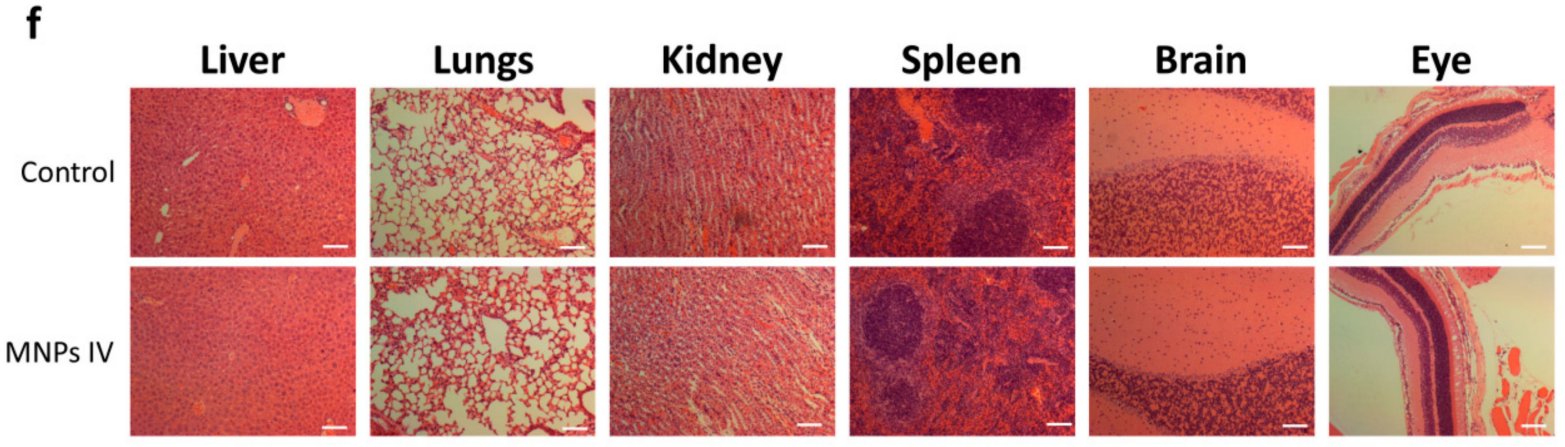
(**a**) A schematic drawing to illustrate in vivo magnetic targeting with intravenous injection; (**b**) Fluorescent and BF images by Maestro imaging of extracted retinas: untreated retina (left), retina close to an external magnet (middle) and retina opposite to magnet side (right); (**c**) Quantitative analysis of fluorescence image shown in (**b**); (**d**) Light microscopy image showing the Prussian blue iron staining followed by counterstaining with nuclear fast red of targeted eye. Scale bar = 100 µm; (**e**) Biodistribution of particles in mice after magnetic targeting. Inductively coupled plasma (ICP) quantification of iron in organs collected from three test groups: untreated control mice, injected mice and magnet targeted injected mice; (**f**) Images of H&E stained organs collected from control and MNPs injected mice 18 days after injection.

Another therapeutic route that is less invasive is the administration of drugs intravenously (i.v.). Such treatment is systemic and it relies on passive accumulation in target sites. Here we demonstrate the magnetic targeting approach for directing the magnetic carriers, through the blood stream, to a selected site. As NGF plays an important therapeutic role in retinal pathologies [61,62] we aimed to target NGF-MNPs towards the mice retina. Mice were i.v. injected with NGF-MNP solution (4 mg/mL, 100 µL) and a magnet was immediately placed near one of the eyes (right eye, Figure 7a). The mice were then sacrificed and the retinas from both eyes were extracted and imaged by in vivo fluorescence imaging system (Maestro, see methods). Retinas from both sides were compared to retinas of untreated mice. Remarkably, as seen in Figure 7b, the fluorescence intensity emitted from the retina of the magnet side was significantly higher than the fluorescence from the opposed retina that was not activated magnetically. Quantitative analysis of fluorescence imaging data revealed that the magnetic targeting enhanced the MNPs fluorescence in the retina region by 2.5 fold (Figure 7c). The significant difference in fluorescence intensity indicates the enrichment of NGF-MNPs in a targeted region when compared to non-targeted sites. Prussian blue staining images demonstrate the presence of MNPs in the magnet targeted eye (Figure 7d).

To validate the possible side effects and therapeutic relevance of the NGF-MNPs, we examined the whole-body biodistribution of the carriers following the treatment. Healthy mice were administrated with NGF-MNPs at a dose of 20 mg/kg intravenously and compared to control mice. Organ samples were collected and dissolved and the iron amount was quantified. ICP measurements indicate that the accumulation of MNPs in eyes at magnet site (right eye) was ~2-fold higher when compared to the passive accumulation of nanoparticles in non-targeted eyes (Figure 7e). A significant accumulation of nanoparticles was observed in the liver and spleen, as these organs play a role in clearance, similar to the bio-distribution profiles of the majority of other nanomaterials that are commonly used in biomedical applications [63,64]. No evidence of MNPs was found in brain and lungs. The body weights of the mice were recorded within the whole evaluation period of 18 days. No body weight loss was observed for the injected mice, showing that the administration of NGF-MNPs has no systemic influence. Histopathological images of liver, lung, kidney, spleen, brain, and eye show no observable pathological abnormalities, suggesting that the MNPs did not induce any damage to treated mice (Figure 7f). These preliminarily in vivo evaluations demonstrate the biocompatibility of NGF-MNPs for possible biomedical applications.

To translate the magnetic approach to other tissue treatments some physical considerations should be discussed. The use of external magnets imposes serious limitations in targeting deep tissues as their field strength and field gradient decrease exponentially with the distance from the outer surface [65]. In order to overcome this challenge, it has been proposed to implement magnetic systems that are based on a combination of external magnets and magnetic devices implanted locally at the target region [66]. The external magnet typically provides the magnetizing field for the carrier, while the local magnet provides the gradients for targeting. This approach has been demonstrated in blood vessels where magnetizable stents were placed for the targeting of endothelial cells and drugs [67,68,69,70].

The fact that MNPs not only serve as a magnetic drug carrier that can be remotely guided, but can also serve as contrast agents for MRI, enabling the monitoring of treated areas, demonstrates the dual modality of MNPs opening possibilities toward theranostics.

## 4. Conclusions

In summary, we demonstrated the ability to direct magnetic nano-carriers loaded with growth factors to specific target sites within the nervous system. Using the device we developed, a modular actuation of magnetic fields can be generated and be adapted to direct MNPs according to the desired application. We successfully differentiated selected populations of cells in culture by directing MNPs conjugated to growth factors to these populations solely. We showed the ability to guide these carriers also in vivo by an external magnetic field, leading to an accumulation at the magnet site. The specific MNPs we have used are favorable as they are stable, biocompatible and penetrate efficiently into tissues and cells. Moreover, MNPs can be used as imaging contrast agents presenting a dual modality characteristic. Together with the ability to control MNP distribution remotely by pre-designed modular magnetic fluxes, this system is advantageous and suitable for novel regenerative therapeutics.

## Supplementary Materials

The following are available online at http://www.mdpi.com/2079-4991/8/9/707/s1.
