# Supplementary material for: Magnetic Targeting of Growth Factors Using Iron Oxide Nanoparticles
Source: Nanomaterials (Basel). 2018 Sep 10;8(9):707. doi: 10.3390/nano8090707 (PMC6163445; doi:10.3390/nano8090707)
Supplement: Supplementary file 1 [file nanomaterials-08-00707-s001.pdf]

Supplementary

Supplementary 1: Bare MNPs, before the conjugation to NGF, present a hydrodynamic diameter of 45 ± 17, as determined by dynamic light scattering.

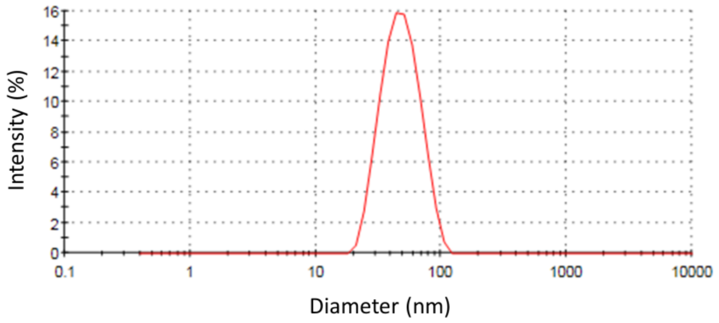

Figure S1. Dynamic light scattering measurement of bare MNPs hydrodynamic diameter.

Supplementary 2: Using COMSOL software, we simulated the magnet field gradient above the device with 6 magnet rods in a form of a circle with a single rod in the center in the opposite direction.

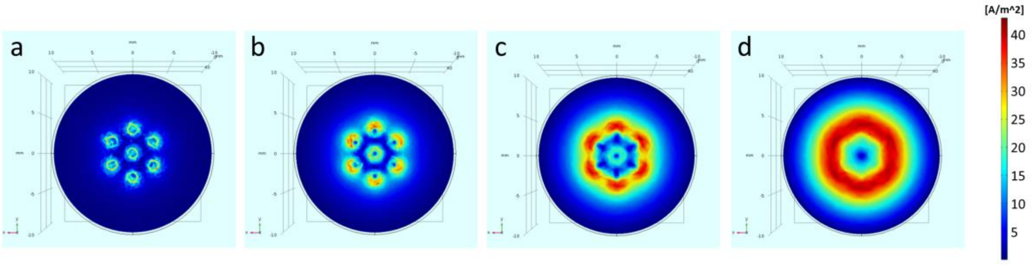

Figure S2. Simulation of magnetic field gradient (a) 0.1mm from top (b) 0.5mm from top (c) 1mm from top (d) 2mm from top.

Supplementary 3: In order to manipulate MNPs, a magnetic field gradient is required to exert a force at a distance. The force on a magnetic nanoparticle with magnetic moment $\vec{m}$ is governed by the equation:[27]

(1) $$F_m = (m_p \cdot \nabla)B$$

where m_p is the magnetic moment of the particle and B is the magnetic field flux density. Due to the superparamagnetic properties of the particles, the magnetic moment is proportional to the external field

(2)

$$m_p = \frac{V_m \Delta\chi B}{\mu_0}$$

where V_m is the volume of the particle and χ is the magnetic susceptibility of the particle. Hence, equation (1) becomes

$$F_m = \frac{V_m \Delta\chi}{\mu_0} (B \cdot \nabla) B \quad (3)$$

To maximize the force, the magnet system should, on the one hand, generate field $\vec{B}$ that is sufficiently strong at the location of the magnetic nanoparticle to maximize the induced magnetization m_p. On the other hand, the magnet system should generate strong field gradients at the nanoparticle's location.
